# Characterization and Properties of Mg-xGd-1.5Nd-0.5Zn-0.5Zr Alloys for Biodegradation Applications

**DOI:** 10.3390/ma13061421

**Published:** 2020-03-20

**Authors:** Zhenzhen Gui, Junyi Zhang, Zhixin Kang

**Affiliations:** 1 School of Mechanical and Electrical Engineering, Guangzhou University, 230 Wai Huan Xi Road, Guangzhou Higher Education Mega Center, Guangzhou 510006, China; zhenzhengui@gzhu.edu.cn; 2 Guangdong Key Laboratory for Advanced Metallic Materials Processing, School of Mechanical & Automotive Engineering, South China University of Technology, 381 Wushan Road, Guangzhou 510640, China; zhangjy@fastengk.com

**Keywords:** magnesium alloys, microstructure, mechanical properties, corrosion resistance

## Abstract

The differences in microstructural characteristics, mechanical properties, and corrosion behavior of the as-cast and solution-treated Mg-xGd-1.5Nd-0.5Zn-0.5Zr alloys (Mg-xGd, x = 1, 3, and 5) were studied and discussed. The as-cast Mg-xGd alloys mainly consisted of an α-Mg and island-like eutectic (Mg,Zn)_3_RE phase, a few cuboidal phases (REH_2_), and a ZnZr phase. With the increase of Gd content, the grain sizes of the as-cast Mg-xGd alloys decreased. Compared to the microstructure of the as-cast Mg-xGd alloys, the eutectic (Mg,Zn)_3_RE phase disappeared and the cuboidal REH_2_ phases existed in the solution-treated Mg-xGd alloys. A large amount of ZnZr_x_ phase was precipitated from α-Mg in the Mg-3Gd alloy and demonstrates a flower-like distribution. The ultimate tensile strength (UTS) and yield strength (YS) of the solution-treated Mg-xGd alloys increased with an increasing Gd content, with the UTS and YS of the Mg-5Gd alloys reaching 217.5 and 125.2 MPa, respectively. Immersion and electrochemical tests showed that the as-cast Mg-3Gd alloy presented the best corrosion resistance with a corrosion rate of 0.285 mm/yr. The corrosion resistance of the solution-treated Mg-3Gd alloy attained the lowest value (0.973 mm/yr), due to the large quantities of ZnZr_x_ with a flower-like phase distribution, forming series of galvanic couple groups with the α-Mg.

## 1. Introduction

Magnesium for biomedical applications has received considerable attention due to its good biodegradation, non-toxicity, and biocompatibility [1,2,3,4]. Mg that is utilized in biodegradable implanting materials needs to provide an appropriate structural support (good mechanical properties) [5,6] and have a suitable dissolving/degradation process (low corrosion rate [7,8,9] and suitable corrosion process) without being harmful to humans (a suitable quantity of non-toxic elements). Erinc [10] suggested that available Mg alloys for biodegradable implants application should achieved the following properties: 1) the corrosion rate in simulated body fluid (SBF) needs to be < 0.5 mm/year (mm/yr); 2) the yield strength (YS) > 200 MPa and the elongation (EL) > 15%. To affect the mechanical properties and corrosion resistance of pure magnesium, alloying and heat treatments are the most effective and inexpensive methods [11,12].

Al is reported to enhance the mechanical properties of Mg-Al (denoted as AZ) series alloys, such as the AZ31 (Mg-3Al-Zn), AZ61 (Mg-6Al-3Zn), and AZ91D (Mg-9Al-Zn) [13] alloys. However, excessive doses of Al in humans may be associated with a possible rise of Alzheimer disease [14], which limits the application of the AZ series magnesium alloys as implant materials [1]. When choosing the alloying elements, their cytotoxicity and content should be taken into consideration [15]. A small quantity of Zn has a certain effect on the grain refinement [16]. An appropriate content of Zr is routinely added as a grain refining element and impurity removal reagents [8]. Rare earth (shortened to RE, such as Gd and Nd) elements not only improve the mechanical properties of magnesium alloys, but also improve several unique performances of Mg alloys [15,17]. This results from good solid solubility of RE elements and induced precipitations in the Mg matrix, thus contributing to solution strengthening and precipitation strengthening [18,19]. Adding RE to form magnesium alloys generally enhances their corrosion properties, due to the formation of a protective oxide film, RE_2_O_3_ [20,21,22]. Considering that the addition of these elements results in better mechanical properties than those of pure magnesium, Mg-RE-Zn alloys have been widely investigated [17,23,24]. Liu et al. [17] found that most of the Mg-RE alloys, such as Mg-1Gd, improved the ultimate tensile strength (UTS, to the value around 213 MPa), yield strength (YS, to the value around 180 MPa), elongation (EL, to the value around 20.5%) and corrosion resistance (to the value around 0.4 mm/yr for immersion 72 h) of pure Mg. Mao et al. [23] showed that extruded Mg–Nd–Zn–Zr (wt.%: 3.09Nd, 0.22Zn, 0.44Zr, and balance Mg) alloy displayed good corrosion resistance with a corrosion rate of 0.337 mm/yr while that of the WE43 alloy with similar extrusion process was 0.714 mm/yr. Among those rare earth elements, a high solid solution of Gd in the Mg matrix improved the solution strength [25], and the corrosion potential of Nd—being similar to that of Mg—reduced the galvanic corrosion tendency in Mg-RE alloys [4], making Gd and Nd the most studied elements in Mg-RE alloys. The quantities of elements in alloys play an important role, affecting their application as biomedical implant materials. Zhang et al. [24] studied the mechanical properties and corrosion behavior of Mg-2Zn-0.2Mn-xNd with various Nd contents, and found that the UTS is significantly improved by Nd addition, due to refinement and second-phase strengthening. Researchers also showed a wide interest in the effects of heat treatment on magnesium alloys. Janbozorgi et al. [11] studied the mechanical and corrosion properties of Mg–2Zn–1Gd–1Ca (wt.%) of as-cast and different heat-treated conditions and found that precipitations improved the ultimate shear strength up to 32%, due to the aging treatment. Prabhu et al. [12] found that the corrosion resistance of Mg-4Zn alloy increased with solution heat treatment, but decreased with precipitation heat treatment. These studies indicate that heat treatment is a promising technique, to alter the mechanical and corrosion properties of Mg-based alloys.

In summary, designing a series of magnesium alloys with suitable alloying elements and investigating how heat treatments and element contents affect the mechanical properties and corrosion rates will help to promote the application of magnesium alloys as biodegradable materials. In this paper, Gd and Nd were chosen as the primary alloying elements to enhance the mechanical properties and corrosion resistance. Zn and Zr were chosen as micro elements to refine the grain size. Mg-xGd-1.5Nd-0.5Zn-0.5Zr alloys (referred to as Mg-xGd, x = 1, 3, and 5) were prepared with casting and heat treatment. The effects of alloy conditions and Gd contents on the microstructure, mechanical properties and corrosion properties were investigated to identify the optimum alloy composition, exhibiting desirable properties.

## 2. Materials and Methods

The Mg-xGd-1.5Nd-0.3Zn-0.3Zr (hereinafter wt.%, shortened as Mg-xGd) alloys were prepared by gravity casting with raw materials consisting of pure Mg (99.9%), pure Zn (99.9%), and master alloys: Mg-25%Gd, Mg-30%Nd, and Mg-25%Zr. The detailed casting process is described in our previous study [26]. The casting ingot was conical-shaped, its diameter ranged from 50 to 60 mm, and its height was 100 mm. The compositions of the as-cast Mg-xGd ingots are shown in Table 1, and were named as Mg-1Gd, Mg-3Gd, and Mg-5Gd, respectively. The as-cast alloy was homogenized at 540 °C for 16 h in a vacuum furnace with a vacuum pressure of 10^−2^ Pa, and then quenched in water at room temperature. These heat-treated ingots are herein referred to as the solution-treated ones.

All samples for testing were cut from the center of the Mg-xGd billets. Tensile tests were conducted on a SANSCMT 5105 universal testing machine with a strain rate of 1.0 × 10^−3^ s^−1^ at room temperature. Microstructure observation of the samples was investigated by optical microscopy (Leica, DMI 5000, Wetzlar, Germany), and an scanning electron microscope (SEM) (Pheom proX, Phenom-World B.V., Eindhoven, Netherlands) equipped with energy-dispersive X-ray spectrometry (EDS). X-ray Diffraction (XRD, D8 ADVANCE, Burker, Bremen, Germany) analysis was utilized to analyze the phases of the samples.

To evaluate the corrosion properties of the studied Mg-xGd alloys, static immersion tests were conducted in Hanks’ solution. The compositions of the Hanks’ solution are listed in Table 2 [27]. The surface area of the samples used for the immersion tests were 10 cm^2^. Each side of the samples was ground up to 2000 grit utilizing series sand papers, and then cleaned with ethanol and distilled water, and dried in a stream of warm air. The ratio of volume of Hanks’ solution (mL) to specimen surface (cm^2^) was 20:1. The immersion tests were conducted in Hanks’ solution for 120 h at 37 °C. After the weight loss experiments, the samples were removed and cleaned in a detergent in an 80 °C water bath for 5 min to observe the corroded surfaces.

An electrochemical workstation (IM6ex, Zahner, Kronach, Germany) and a three-electrode cell with a platinum electrode as the counter electrode, samples as the working electrode, and a saturated calomel electrode (SCE) as the reference electrode were utilized to study the electrochemical properties. Samples were immersed in the Hanks’ solution at the open circuit potential (OCP) for 30 min to evaluate the change of potential. After the potential achieved a constant value, potentiodynamic polarization test was undergo at a scanning rate of 5 mV/s. The voltage span for potentiodynamic polarization testing was −5 mV to 5 mV on the basis of the constant potential obtained through the OCP test. Three specimens were evaluated for each alloy.

The average corrosion rates were calculated through weight loss after immersion tests (*P*_W_) or the hydrogen evolution tests (*P*_H_), by the following Equations (1) and (2) [27]:

(1) $$P_{W}=\frac{87600\Delta W}{AT\rho }$$

*P*_H_ = 2.088*V*_H_ (2)

Δ*W*(g) is the weight loss during the immersion test, *A* (cm^2^) is the contact area of the samples with solution, *T* (h) is the testing time, *ρ* is the density of Mg-xGd alloys, and *V*_H_ (mL·cm^−2^·d^−1^) is the rate of hydrogen evolution.

## 3. Results

### 3.1. Microstructure of Mg-xGd Alloys

**Figure 1:**
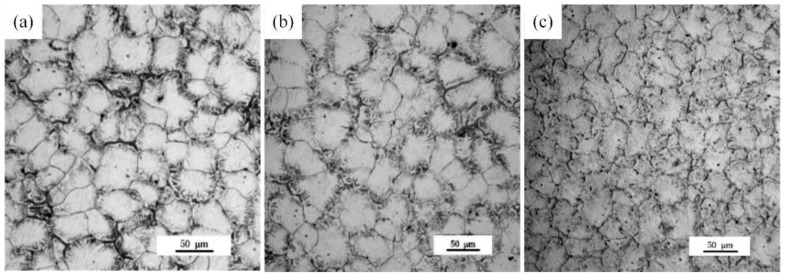
Optional microstructure of the as-cast Mg-xGd alloys: (**a**) Mg-1Gd (AC (as-cast)), (**b**) Mg-3Gd (AC), and (**c**) Mg-5Gd (AC)**.**

Grains of the as-cast (referred to as AC) Mg-xGd alloys mostly exhibit an equiaxed morphology, as shown in Figure 1. Grain sizes of the Mg-xGd alloys with different Gd contents were measured through the linear intercept method, with values of 52.3, 49.2, and 40.9 μm, respectively, obtained. Grain growth was restrained due to the addition of Gd during the melting solidification process. A large quantity of eutectic phases was distributed around the grain boundaries. A slight granular phase was present in the as-cast Mg-xGd grains.

**Figure 2:**
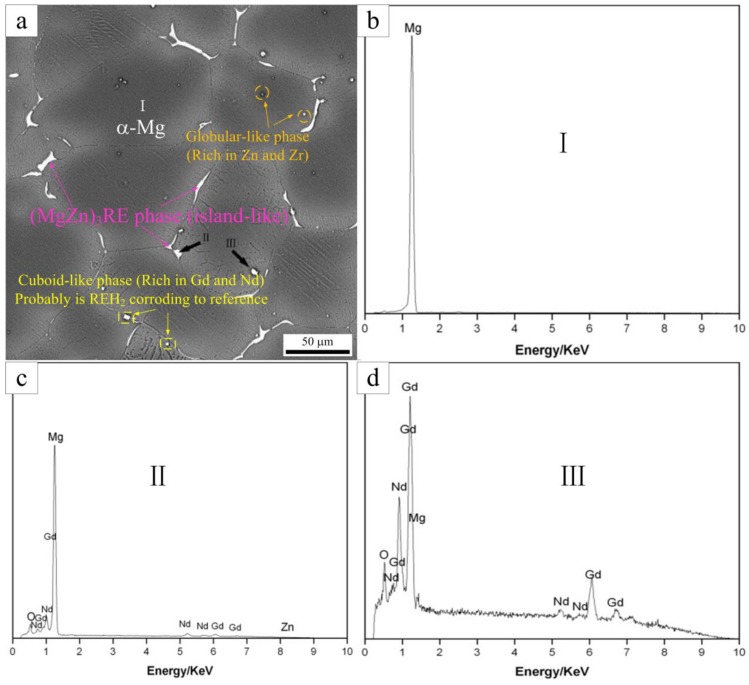
The SEM image of the as-cast Mg-5Gd alloy (**a**), the EDS result of point “I” (**b**), the EDS result of point “II” (**c**), and the EDS result of point “III” (**d**).

To further investigate the phases present in the as-cast Mg-5Gd alloy, SEM analysis was carried out, as shown in Figure 2. Four kinds of phases in the as-cast Mg-5Gd alloy were shown to exist, as presented in Figure 2a: One phase was the matrix α-Mg (as marked at position “I”), one phase was the island-like eutectic phase distribution around grain boundaries (as marked at position “II” and “III”), one phase was a globular-like ZnZr_x_ phase (as marked in circles), and one phase was cuboid-like, rich in Gd and Nd, which most likely was REH_2_ (as marked in rectangles) according to [28]. EDS analysis of points “I”, “II”, and “III” was carried out as shown in Figure 2b–d). The EDS results are shown in Table 3. According to the EDS results, the island-like eutectic phase was most likely Mg_3_Gd, which was formed according to the eutectic equation L→α-Mg + Mg_3_Gd during non-equilibrium solidification [29]. As a result of the addition of Nd and Zn, some Gd and Mg atoms were replaced by Nd and Zn atoms, therefore, the Mg_3_Gd phase was transformed into the (Mg,Zn)_3_RE phase. A similar phenomenon was found in [30].

**Figure 3:**
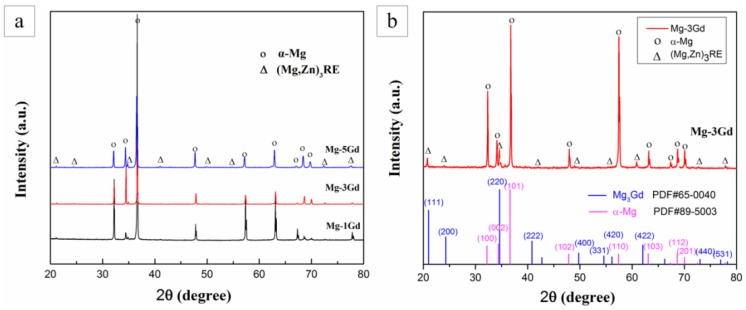
The X-ray Diffraction (XRD) pattern of the as-cast Mg-xGd alloys (**a**), phase comparison and corresponding ICCD cards (**b**).

To obtain a further understanding of the phases present in the Mg-xGd alloys, XRD analysis was carried out, as shown in Figure 3a. The Mg-xGd alloys mainly consisted of α-Mg peaks according to the standard ICCD (International Center for Diffraction Data) card: PDF#89-5003, as displayed in Figure 3b. The other displayed peaks in accordance with the Mg_3_Gd-type phase according to the standard peaks of (PDF#65-0040). A small migration in 2θ was due to the solid solution of Nd and Zn in the Mg_3_Gd phase; therefore, it was identified as the (MgZn)_3_RE phase. The XRD results were in accordance with the above EDS results. The globular-like ZnZr_x_ phase and cuboid-like REH_2_ phase in SEM did not appear in the diffraction peaks as their total amounts were too few to be identified by the XRD analysis.

**Figure 4:**
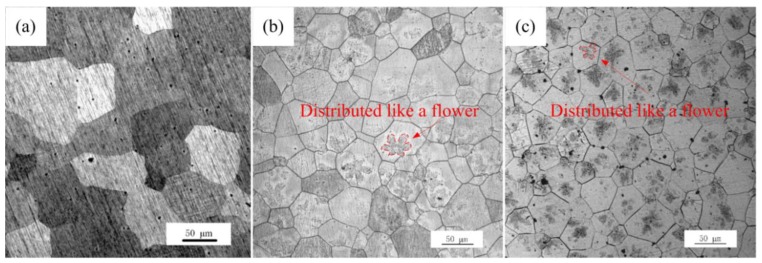
Microstructure of the solution-treated (ST) Mg-xGd alloys: (**a**) Mg-1Gd (ST), (**b**) Mg-3Gd (ST), and (**c**) Mg-5Gd (ST).

Microstructures of the Mg-xGd alloys were different between the as-cast alloys and the solution-treated (referred to as ST) alloys. Firstly, the grain sizes of the two kinds of the as-cast and solution-treated alloys are different. The microstructures of solution-treated Mg-xGd alloys with different Gd contents are shown in Figure 4. The grains of the solution-treated samples generally grew to 68.5, 55.6, and 48.2 μm, respectively. Compared to the as-cast Mg-xGd alloys, the grain sizes of the solution-treated Mg-xGd alloys increased by 31%, 13%, and 18%. Among which, Mg-1Gd demonstrated an evident increase due to having too few eutectic phases to restrain its growth. Then, the morphology of the phases changed after solution treatment. Most elements of the original eutectic phases in the as-cast condition were solid solution into the α-Mg matrix. Only part of the cuboid-like phase still existed in the solution-treated alloys. New phases were precipitated in the solution-treated alloys. A large amount of irregular precipitates distributed like flowers precipitated from the α-Mg matrix in the solution-treated Mg-3Gd and Mg-5Gd samples, as shown in Figure 4b,c. A similar phenomenon was found in [31], and this contributed to the precipitation of the ZnZr_x_ phase during the solution-treating process.

**Figure 5:**
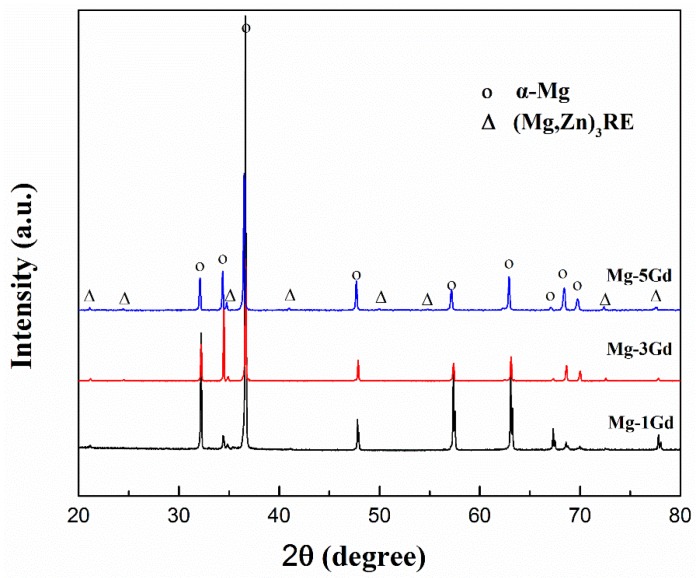
The XRD pattern of Mg-xGd alloys after solution treatment.

For further elucidation of the phase transformations of the solution-treated Mg-xGd alloys, XRD analysis of the solution-treated Mg-xGd alloys was carried out. The XRD results of the solution-treated samples are shown in Figure 5. The intensities of the diffraction peaks of the eutectic phase (Mg,Zn)_3_RE decreased dramatically in the solution-treated Mg-5Gd alloy, compared to that in the as-cast Mg-5Gd alloy. Certain diffraction peaks of the eutectic phase (Mg,Zn)_3_RE completely disappeared in the solution-treated Mg-3Gd alloy, compared to the as-cast Mg-3Gd alloy. This result indirectly indicated that the eutectic phase (Mg,Zn)_3_RE was incorporated into the α-Mg matrix.

### 3.2. Mechanical Properties of the Mg-xGd Alloys

**Figure 6:**
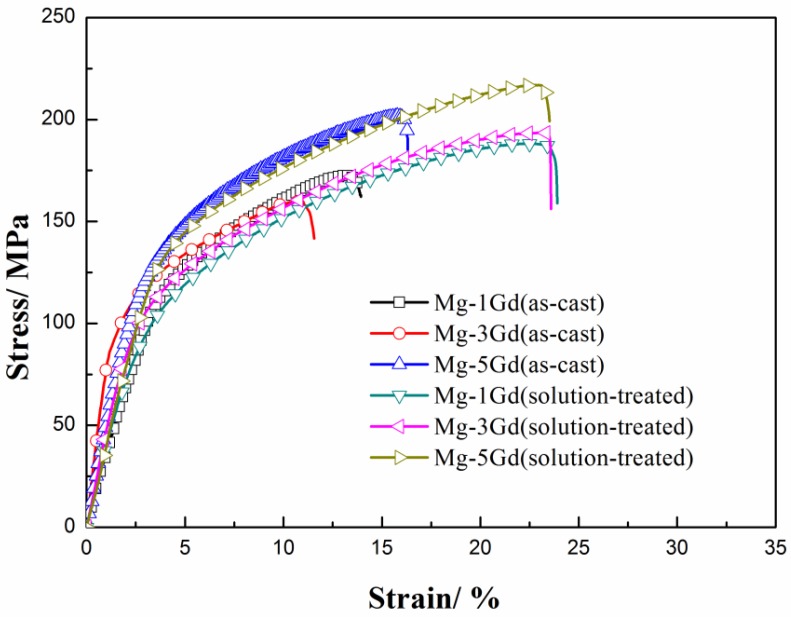
The stress–strain curves of the Mg-Gd alloys under different conditions.

The tensile stress versus strain curves of the studied Mg-xGd alloys are depicted in Figure 6. The UTS, YS, and tensile fracture elongation (shortened as EL) obtained from these curves are also listed in Table 4. The as-cast Mg-3Gd alloy presented the lowest mechanical properties values: with UTS at 160.0 MPa, YS at 85.8 MPa, and EL at 11.2%—among the three as-cast Mg-xGd alloys. The UTS and YS of the as-cast Mg-3Gd alloy were enhanced by 21.5% and 30%, respectively, through the solution treatment. The UTS and YS of the Mg-xGd alloys increased with increasing Gd content after solution treatment, and the solution-treated Mg-5Gd alloy showed the highest values, with UTS at 125.2 MPa and YS at 217.5 MPa.

### 3.3. Immersion Tests of the Mg-xGd Alloys

**Figure 7:**
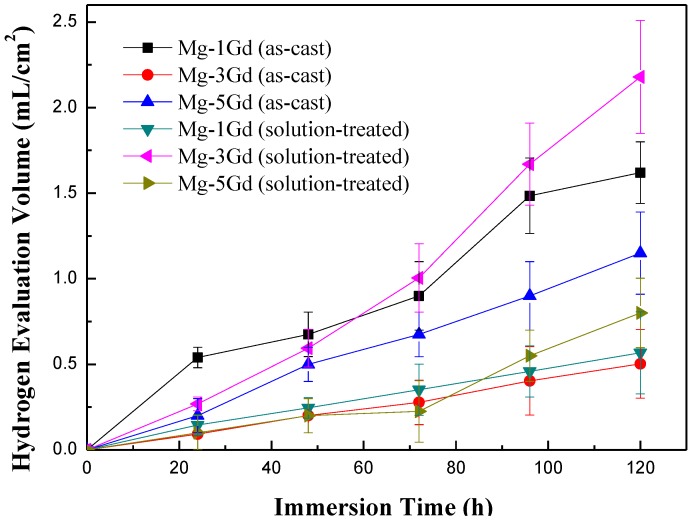
The hydrogen evolution curves of the Mg-xGd alloys under different conditions based on static immersion tests.

Hydrogen evolution curves of the as-cast and solution-treated Mg-xGd alloys were recorded during an immersion test in Hanks’ solution for 5 days, as shown in Figure 7. The hydrogen evolution volume increased with the immersion time. The rate of hydrogen evolution fluctuated within a narrow range during the immersion period. The results of the corrosion rate preliminarily displayed that Mg-3Gd (ST) > Mg-1Gd (AC) > Mg-5Gd (AC) > Mg-5Gd (ST) > Mg-1Gd (ST) > Mg-3Gd (AC).

**Figure 8:**
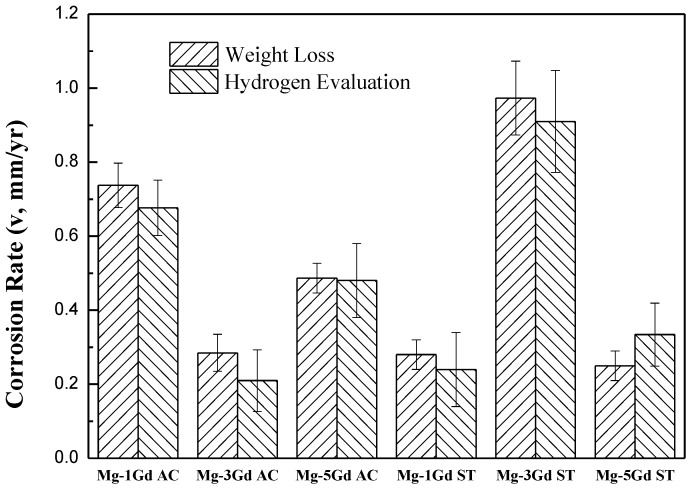
Corrosion rates of the as-cast, solution-treated Mg-xGd alloys in Hanks’ solution for 5 days at 37 °C.

Corrosion rates of the Mg-xGd alloys were calculated according to Equations (1) and (2) as shown in the former part. The calculated results are displayed in Figure 8. The lowest corrosion rate appeared in the Mg-3Gd (AC) sample, which was 0.285 ± 0.050 mm/yr, while the Mg-3Gd (ST) sample presented the highest value of corrosion rate of 0.973 ± 0.100 mm/yr. These results indicated that Mg-3Gd (AC) shows a good corrosion resistance (<0.5 mm/yr) among the studied as-cast Mg-xGd alloys.

**Figure 9:**
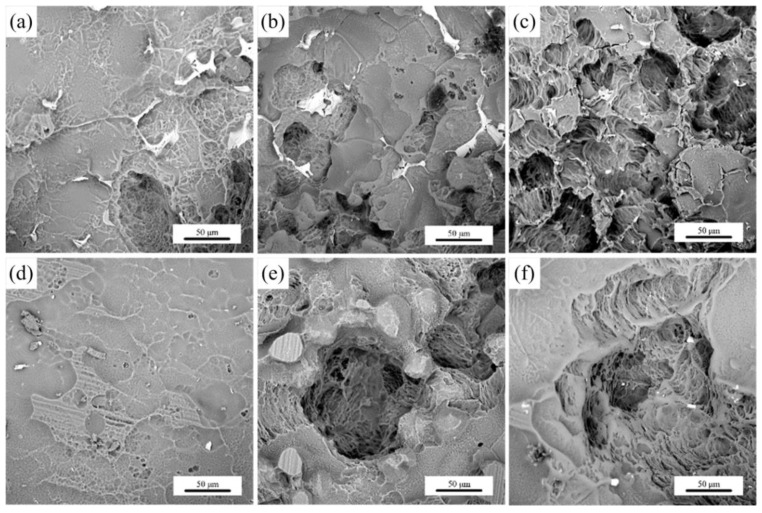
Surface morphologies of Mg-xGd alloys after surface corrosion: (**a**) Mg-1Gd (AC), (**b**) Mg-3Gd (AC), (**c**) Mg-5Gd (AC), (**d**) Mg-1Gd (ST), (**e**) Mg-3Gd (ST), and (**f**) Mg-5Gd (ST).

To elucidate the corrosion mechanism of Mg-xGd alloys, the surface morphologies of the corroded Mg-xGd alloys after the removal of corrosion products were obtained through SEM. Most regions of the corroded surface of the Mg-xGd alloys were well protected by oxidation films. The seriously corroded surface regions of the Mg-xGd alloys are displayed in Figure 9. The as-cast Mg-xGd alloys presented intergranular corrosion modes. The corrosion process appeared initially in the α-Mg matrix around grain boundaries, due to micro galvanic corrosion forming between the α-Mg matrix (as the anode) and eutectic (Mg,Zn)_3_RE (as the cathode). Then, the corrosion process expanded to inside the α-Mg matrix and finally formed corrosion pits. The size and depth of the corrosion pits were used to evaluate the corrosion resistance of the as-cast Mg-xGd alloys. The Mg-3Gd (AC) alloy displayed the highest corrosion resistance, followed by the Mg-1Gd (AC) alloy and then, the Mg-5Gd (AC) alloy, as displayed in Figure 9a–c. After being solution-treated, the Mg-1Gd alloy presented a relatively good corrosion resistance, while the Mg-3Gd (ST) and Mg-5Gd (ST) possessed more serious corrosion pits compared to the as-cast Mg-xGd alloys.

## 4. Discussion

### 4.1. The Effection Factors on Mechanical Properties

**Figure 10:**
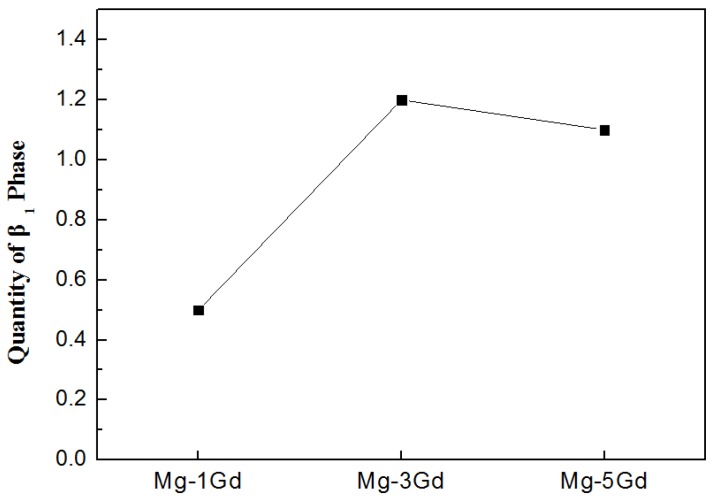
The quantity of the (Mg,Zn)_3_RE phase in the as-cast Mg-xGd alloys based on the XRD pattern.

The UTS and YS of the as-cast Mg-xGd alloys did not increase with the Gd content. The Mg-3Gd alloys presented the lowest values of UTS, YS, and elongation compared with other Mg-xGd alloys in the as-cast condition. Depending on the XRD pattern, the (Mg,Zn)_3_RE phases during the casting were roughly estimated by the WPF method (whole pattern fitting and Rietveld refinement) [32]. As can be seen in Figure 10, the quantity of the (Mg,Zn)_3_RE phases in the as-cast Mg-3Gd alloy was greater than the other as-cast Mg-xGd alloys. Large quantities of the (Mg,Zn)_3_RE phase around the grain boundaries intensified the dendritic segregation and stress concentration simultaneously [26], which decreased the UTS and YS of the as-cast Mg-xGd alloys. After solution treatment, the (Mg,Zn)_3_RE phase dissolved into the α-Mg matrix, which eliminated the negative effect of the eutectic (Mg,Zn)_3_RE phase on the plasticity. The dissolved atoms in the solution-treated Mg-xGd alloys resulted in solution strengthening. The solution strengthening effect increased with increasing Gd content, inducing the UTS and YS of the solution-treated Mg-xGd alloys to synchronously increase with the Gd content. Owing to the solution strengthening effect, the Mg-xGd alloys display better mechanical properties in the solution treated condition compared to those of the as-cast condition. However, the mechanical properties of the Mg-1Gd alloy decreased after solution treatment. This is because the grains of the solution-treated Mg-1Gd alloy dramatically increase in size to 31% greater than those of the as-cast Mg-1Gd. When grains increase in size, the grain boundaries decrease, which leads to a decrease in the grain boundary strengthening. The degree of grain growth of the Mg-3Gd and Mg-5Gd alloy is 13% and 18% after solution treatment. Therefore, that the effect of the grain boundary strengthening on the Mg-3Gd and Mg-5Gd alloy is less than that of the Mg-1Gd alloy.

In summary, the addition of Gd, Nd, and Zn contributes to forming the (Mg,Zn)_3_RE phases as well as effecting the grain size of the as-cast Mg-xGd alloys according to the microstructure. The (Mg,Zn)_3_RE phase in the as-cast Mg-xGd alloys distributed around the grain boundaries and played a negative effect on the mechanical properties due to the dendritic segregation and stress concentration. The addition of Zn and Zr contributes to forming the ZnZrx phase, the amount of ZnZrx is a few in the as-cast Mg-xGd alloys, while a lot of ZnZrx phase precipitated from the matrix during the solution-treating process, which acted as a positive effect on the mechanical properties due to the precipitation strengthening.

### 4.2. The Effect of Gd Content on the Corrosion Properties of the Mg-xGd Alloys

The immersion tests demonstrated that the corrosion resistance of the Mg-3Gd alloy was completely different from those of the other studied Mg-xGd alloys. While according to the previous studies [33,34,35], the as-cast and solution-treated Mg-RE alloys presented better corrosion resistance with increasing Gd content, which was in contrast to the research results found in this paper.

**Figure 11:**
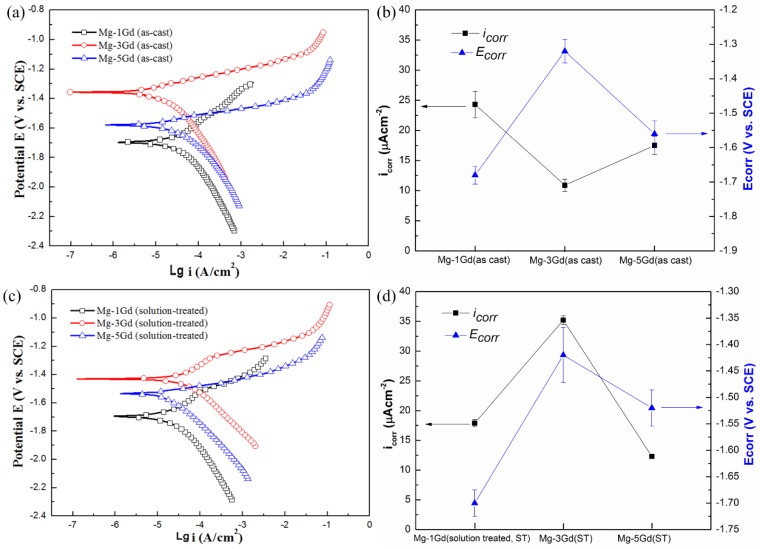
Potentiodynamic polarization curves and corrosion characteristics of different Mg-xGd alloys tested in 37 °C Hanks’ solutions: (**a**) and (**b**) as-cast and (**c**) and (**d**) solution-treated.

To obtain a better understanding of the corrosion properties of different Mg-xGd alloys, potentiodynamic polarization and corrosion characteristics of the as-cast and solution-treated Mg-3Gd alloys were investigated, as shown in Figure 11. In the as-cast samples, the corrosion potential of Mg-3Gd (AC) was −1.319 V, which is higher than Mg-1Gd (AC) (−1.700 V) and Mg-5Gd (AC) (−1.549 V). This demonstrated that, compared with other as-cast Mg-xGd alloys, Mg-3Gd (AC) was not easily corroded in Hanks’ solution. The Mg-3Gd (ST) presented the lowest value of the corrosion potential, and was the easiest to corrode among the three solution-treated Mg-xGd alloys. The corrosion current density (*i*_corr_) of Mg-3Gd (AC) obtained the lowest value (10.7 μA/cm^2^) and that of the Mg-3Gd (ST) obtained the highest value (36.1 μA/cm^2^). This indicates that the as-cast Mg-3Gd corroded the slowest. In other words, the as-cast Mg-3Gd showed the highest corrosion resistance in Hanks’ solution. The results of potentiodynamic polarization curves were in accord with the corrosion rates calculated through the immersion tests.

On account of other researchers’ former work on the corrosion properties of Mg-RE alloys, grain size, variety and microstructure of the second phase (usually referred to as the eutectic phase and precipitated phase, such as the (Mg,Zn)_3_Gd phase and ZnZr_x_ phase) played an important role in the corrosion resistance. Song et al. [36] found that grain refinement resulting from ECAP (equal channel angular pressing) process played two-sided effects on the corrosion properties of the magnesium alloy. On the one hand, fine grains induced more corrosion pits and evident filiform-like corrosion, on the other, the fine grains and uniformly distributed second phase can decrease the affection of galvanic corrosion and inhibit the pitting corrosion [37].

Among the studied Mg-xGd alloys, although the grain size of Mg-xGd alloys decreased with Gd content, the grain size of all of the Mg-xGd alloys was of the same order of magnitude, and the impact of grain size on the corrosion resistance was low. The observed differences in the corrosion properties in the studied Mg-xGd alloys are due to the second phases, which include a eutectic phase and precipitates phase. The influence of the second phase was relatively complex. The existence of the second phase inevitably brought about the interaction with the α-Mg matrix. In the Mg-3Gd (ST) sample there existed large amounts of fine precipitated ZnZr_x_ phase (distributed like flowers). Because of large amounts of precipitates that appeared in the solution-treated Mg-3Gd alloy, the precipitates and the α-Mg formed into a series of galvanic couple groups which would exacerbate the corrosion process and lead to the fastest corrosion rate. On the other hand, owing to the presence of the eutectic (Mg,Zn)_3_Gd phase around the grain boundary, the island-like eutectic (Mg,Zn)_3_Gd phase acted as a “wall” to stop corrosion propagation along the grains. The solution-treated Mg-3Gd alloy displayed the largest quantity of eutectic (Mg,Zn)_3_RE phase around the boundary, resulting in the highest value of corrosion rate (lowest value of corrosion resistance) in the Hanks’ solution. The Mg-1Gd (ST) alloy showed a homogeneous corrosion pattern resulting from the disappearance of its microstructural the eutectic (Mg,Zn)_3_RE phase, which failed to form galvanic couples between the eutectic (Mg,Zn)_3_RE phases and around α-Mg.

The authors of Ref. [38] show that the side effects of Gd depending on its presentation: free ion (toxic) and chelated (very low toxicity). The amount of Gd content as well as Gd concentration in vivo make it difference. Myrissa et al. [39,40] found that the corrosion rate in vitro of the raw material Mg-10Gd (Gd, 10 wt.%) is around 0.56 mm/yr and the Mg-10Gd alloy presents Gd concentration below the limit of detection after implanting in the Sprague–Dawley^®^ rats. Giving an extended application, the Mg-3Gd (lower concentration of Gd in the raw materials compared to the Mg-10Gd) in our study with the in vitro corrosion rate of 0.285 mm/yr (lower corrosion rate compared to that of Mg-10Gd in Ref. [39]) should have a lower concentration of Gd in the rats compared to the result in Ref. [40].

## 5. Conclusions

In this paper, the microstructure, mechanical properties (UTS, YS, and EL), and corrosion properties of the as-cast and solution-treated Mg-xGd-Nd-Zn-Zr alloys were studied and the conclusions are listed as follows:

(1) The as-cast Mg-xGd alloys consisted of an α-Mg matrix. The island-like eutectic (Mg,Zn)_3_RE phase was distributed around the grain boundaries, along with the cuboidal phases (REH_2_) and globular-like ZnZr_x_ phase. The grain size of the as-cast Mg-xGd alloys decreased with increasing Gd content. After solution treatment, the eutectic (Mg,Zn)_3_RE phase in the as-cast Mg-xGd alloys solid soluted into the α-Mg matrix. Meanwhile, the fine ZnZr_x_ phase precipitated from the α-Mg matrix and exhibited a flower-like distribution in the solution-treated Mg-xGd alloys.

(2) Mg-3Gd (AC) presented the lowest value of UTS (160 MPa) and YS (85.8 MPa) among the as-cast Mg-xGd alloys, resulting from the fact it exhibited the largest quantity of eutectic (Mg,Zn)_3_RE phases, which enhanced to intensify the dendritic segregation and stress concentration. After solution treatment, UTS and YS of the Mg-xGd alloys increased with increasing Gd content, and UTS and YS of the solution-treated Mg-5Gd reached 217.5 MPa and 125.2 MPa, respectively.

(3) Mg-3Gd (AC) showed the lowest value of corrosion rate among the as-cast Mg-xGd alloys, with a corrosion rate of 0.285 ± 0.050 mm/yr, while the Mg-3Gd (ST) displayed the highest corrosion rate in the solution-treated Mg-xGd alloys (0.973 ± 0.100 mm/yr).

(4) The effect of microstructure, including the quantity of eutectic phases, precipitations and grain sizes on the corrosion rate of the Mg-xGd alloys is integrative.

## Figures and Tables

**Table 1:** Compositions of the as-cast Mg-xGd-Nd-Zn-Zr alloys (wt.%).

	Gd	Nd	Zn	Zr	Fe	Mg
Mg-1Gd	1.25	1.53	0.56	0.23	0.009	balance
Mg-3Gd	2.63	1.60	0.48	0.39	0.009	balance
Mg-5Gd	4.75	1.42	0.59	0.37	0.009	balance

**Table 2:** The compositions of Hanks’ solutions [27].

Composition	Concentration (g/L)
NaCl	8.0
CaCl_2_	0.14
MgCl_2_·6H_2_O	0.1
KCl	0.4
NaHCO_3_	0.35
MgSO_4_·7H_2_O	0.06
KH_2_PO_4_	0.06
Na_2_HPO_4_·12H_2_O	0.06
C_6_H_12_O_6_	1.0
pH	7.4

**Table 3:** Corresponding of the energy-dispersive X-ray spectrometry (EDS) results of points in the as-cast Mg-5Gd alloy in Figure 2a (atom, %).

Position	Gd	Nd	Zn	O	Mg
I	–	–	–	–	100.0
II	18.2	13.0	7.8	4.7	56.4
III	67.2	9.4	–	5.1	18.3

**Table 4:** The mechanical properties of Mg-xGd alloys.

Samples	YS (MPa)	UTS (MPa)	Elongation (%)
Mg-1Gd AC	136.1	172.8	13.2
Mg-3Gd AC	85.8	160.0	11.2
Mg-5Gd AC	103.3	201.9	15.6
Mg-1Gd ST	102.0	188.5	22.0
Mg-3Gd ST	111.7	194.4	22.9
Mg-5Gd ST	125.2	217.5	22.7

## References

[1] Johnston S., Shi Z., Hoe C., Uggowitzer P.J., Cihova M., Loffler J.F., Dargusch M.S., Atrens A. (2018). The influence of two common sterilization techniques on the corrosion of Mg and its alloys for biomedical applications. J. Biomed. Mater. Res. B.

[2] Mao L., Shen L., Niu J.L., Zhang J., Ding W.J., Wu Y., Fan R., Yuan G.Y. (2013). Nanophasic biodegradation enhances the durability and biocompatibility of magnesium alloys for the next-generation vascular stents. Nanoscale.

[3] Atrens A., Song G.L., Cao F., Shi Z., Bowen P.K. (2013). Advance in Mg corrosion and research suggestions. J. Magnes. Alloys.

[4] Zhang X., Yuan G., Mao L., Niu J.L., Fu P.H., Ding W.J. (2012). Effects of extrusion and heat treatment on the mechanical properties and biocorrosion behaviors of a Mg-Nd-Zn-Zr alloy. J. Mech. Behav. Biomed. Mater..

[5] Xia X.S., Chen Q., Zhao Z.D., Ma M.L., Li X.G., Zhang K. (2015). Microstructure, texture and mechanical properties of coarse-grained Mg-Gd-Y-Nd-Zr alloy processed by multidirectional forging. J. Alloys Compd..

[6] Han H.S., Loffredo S., Jun I., Edwards J., Kim Y.C., Seok H.K., Witte F., Mantovani D., Glyn-Jones S. (2019). Current status and outlook on the clinical translation of biodegradable metals. Mater. Today.

[7] Liu W., Yan Z.J., Zhang Z.D., Zhang Y.X., Cai G.Y., Li Z.Y. (2019). Bioactive and anti-corrosive bio-MOF-1 coating on magnesium alloy for bone repair application. J. Alloys Compd..

[8] Zhang X.B., Dai J.W., Zhang R.F., Ba Z.X., Nick B. (2019). Corrosion behavior of Mg-3Gd-1Zn-0.4Zr alloy with and without stacking faults. J. Magnes. Alloys.

[9] He R.G., Liu R.Y., Chen Q.B., Zhang H.J., Wang J.F., Guo S.F. (2018). In Vitro degradation behavior and cytocompatibility of Mg-6Zn-Mn alloy. Mater. Lett..

[10] Erinc M., Sillekens W.H., Mannens R.G.T.M., Werkhoven R.J. (2009). Applicability of existing magnesium alloys as biomedical implant materials. Magn. Technol..

[11] Janbozorgi M., Taheri K.K., Taheri A.K. (2018). Microstructural evolution, mechanical properties, and corrosion resistance of a heat-treated Mg alloy for the bio-medical application. J. Magnes. Alloys.

[12] Prabhu D.B., Nampoothiri J., Elakkiya V., Narmadha R., Selvakumar R., Sivasubramanian R., Gopalakrishnan P., Ravi K.R. (2020). Elucidating the role of microstructural modification on stress corrosion cracking of biodegradable Mg-4Zn alloy in simulated body fluid. Mater. Sci. Eng. C.

[13] Wen Z.H., Wu C.J., Dai C.S., Yang F.X. (2009). Corrosion behaviors of Mg and its alloys with different Al contents in a modified simulated body fluid. J. Alloys Compd..

[14] Bondy S.C. (2016). Low levels of aluminum can lead to behavioral and morphological changes associated with Alzheimer’s disease and age-related neurodegeneration. Neurotoxicology.

[15] Chen Y., Xu Z., Smith C., Sankar J. (2014). Recent advances on the development of magnesium alloys for biodegradable implants. Acta Biomater..

[16] Paramsothy M., Gupta M. (2013). In-Situ rod-shaped nanoparticles in Mg–Zn magnesium alloy: Towards high strength and ductility. J. Alloys Compd..

[17] Liu J., Bian D., Zheng Y., Chu X., Lin Y., Wang M., Lin Z., Li M., Zhang Y., Guan S. (2020). Comparative In Vitro study on binary Mg-RE (Sc, Y, La, Ce, Pr, Nd, Sm, Eu, Gd, Tb, Dy, Ho, Er, Tm, Yb and Lu) alloy systems. Acta Biomater..

[18] Hidalgo-Manrique P., Robson J.D., Pérez-Prado M.T. (2017). Precipitation strengthening and reversed yield stress asymmetry in Mg alloys containing rare-earth elements: A quantitative study. Acta Mater..

[19] Nie J.F., Oh-ishi K., Gao X., Hono K. (2008). Sotute segregation and precipitation in a creep-resistant Mg-Gd-Zn alloy. Acta Mater..

[20] Yan K., Su J., Zhao Y. (2017). Microstructure and mechanical properties of the laser-welded Mg-3Nd-0.2Zn-0.4Zr (NZ30K) magnesium alloy. Opt. Laser Technol..

[21] Wang M., Xiao D.H., Liu W.S. (2017). Effect of Si addition on microstructure and properties of magnesium alloys with Al and Zn contents. Vacuum.

[22] Edalati K., Masuda T., Arita M., Furui M., Sauvage X., Horita Z., Valiev R.Z. (2017). Room-temperature superplasticity in an ultrafine-grained magnesium alloy. Sci. Rep..

[23] Mao M., Yuan G., Wang S., Niu J., Wu G., Ding W. (2012). A novel biodegradable Mg–Nd–Zn–Zr alloy with uniform corrosion behavior in artificial plasma. Mater. Lett..

[24] Zhang Y., Li J.X., Li J.Y. (2018). Microstructure, mechanical properties, corrosion behavior and film formation mechanism of Mg-Zn-Mn-xNd in Kokubo’s solution. J. Alloys Compd..

[25] Arrabal R., Matykina E., Pardo A., Merino M.C., Paucar K., Mohedano M., Casajus P. (2012). Corrosion behavior of AZ91D and AM50 magnesium alloys with Nd and Gd additions in humid environments. Corros. Sci..

[26] Zhang J.Y., Kang Z.X., Zhou L.L. (2015). Microstructure evolution and mechanical properties of Mg-Gd-Nd-Zn-Zr alloy processed by equal channel angular pressing. Mater. Sci. Eng. A.

[27] Atrens A., Song G.L., Liu M., Shi Z., Cao F., Dargusch M.S. (2015). Review of recent developments in the field of magnesium corrosion. Adv. Eng. Mater..

[28] Zheng K.Y., Dong J., Zeng X.Q., Ding W.J. (2008). Precipitation and its effect on the mechanical properties of a cast Mg-Gd-Nd-Zr alloy. Mater. Sci. Eng. A.

[29] Cao F.R., Ding H., Li Y.L., Zhou G., Cui J.Z. (2010). Superplasticity, dynamic grain growth and deformation mechanism in ultra-light two-phase magnesium-lithium alloys. Mater. Sci. Eng. A.

[30] Bamberger M., Atiya G., Khawaled S., Katsman A. (2014). Comparison study of microstructure and phase evolution in Mg-Nd- and Mg-Gd- based alloys. Metall. Mater. Trans. A.

[31] Sha G., Li J.H., Xu W., Xia K., Jie W.Q., Ringer S.P. (2010). Hardening and microstructural reactions in high-temperature equal-channel angular pressed Mg-Nd-Gd-Zn-Zr alloy. Mater. Sci. Eng. A.

[32] Yuan Y.C., Ma A.B., Jiang J.H., Sun Y., Lu F.M., Zhang L.Y., Song D. (2014). Mechanical properties and precipitate behavior of Mg-9Al-1Zn alloy processed by equal-channel angular pressing and aging. J. Alloys Compd..

[33] Hort N., Huang Y., Fechner D., Störmer M., Blawert C., Witte F., Vogt C., Drücker H., Willumeit R., Kainer K.U. (2010). Magnesium alloys as implant materials- principles of property design for Mg-RE alloys. Acta Biomater..

[34] Liu D., Ding Y., Guo T., Qin X.Q., Guo C.G., Yu S., Lin S. (2014). Influence of fine-grain and solid-solution strengthening on mechanical properties and in vitro degradation of WE43 alloy. Biomed. Mater..

[35] Wang H., Estrin Y., Zúberová Z. (2008). Bio-corrosion of a magnesium alloy with different processing histories. Mater. Lett..

[36] Song D., Ma A.B., Jiang J.H., Lin P.H., Yang D.H., Fan J.F. (2011). Corrosion behavior of bulk ultra-fine grained AZ91D magnesium alloy fabricated by equal-channel angular pressing. Corros. Sci..

[37] Song G., Xu Z. (2012). Crystal orientation and electrochemical corrosion of polycrystalline Mg. Corros. Sci..

[38] Adding L.C., Bannenberg G.L., Gustafsson L.E. (2001). Basic experimental studies and clinical aspects of gadolinium salts and chelates. Cardiovasc. Drug Rev..

[39] Myrissa A., Agha N.A., Lu Y., Martinelli E., Eichler J., Szakács G., Kleinhans C., Willumeit-Römer R., Schäfer U., Weinberg A.M. (2016). In vitro and In Vivo comparison of binary Mg alloys and pure Mg. Mater. Sci. Eng. C.

[40] Myrissa A., Braeuer S., Martinelli E., Willumeit-Römer R., Goessler W., Weinberg A.M. (2017). Gadolinium accumulation in organs of Sprague–Dawley^®^ rats after implantation of a biodegradable magnesium-gadolinium alloy. Acta Biomater..

